# Knowledge, Attitudes and Practices on the Use of Botanical Medicines in a Rural Caribbean Territory

**DOI:** 10.3389/fphar.2021.713855

**Published:** 2021-10-27

**Authors:** Tatijana Vujicic, Damian Cohall

**Affiliations:** Faculty of Medical Sciences, The University of the West Indies, Cave Hill, St. Michael, Barbados

**Keywords:** caribbean, drug-herb interaction, botanical, medicine, predictors, survey, medicinal plants

## Abstract

The worldwide use of medicinal plant products has been steadily increasing over the past few decades, whereas the traditional knowledge and practices of these botanical medicines appears to be diminishing. Considering the need to conserve and document these traditions, the objective of this study was to understand the knowledge, attitudes, and practices of people who are familiar with botanical medicines, as well as any factors that may influence the perceptions and behaviours associated with the use of medicinal plants. A previously validated survey instrument assessing knowledge, attitudes, and practices on the use medicinal plants was randomly administered to residents of three rural Barbadian communities. The data was analyzed using descriptive statistics and cross tabulations (Chi-Square Test, Fisher’s Exact Test), with a confidence level of 95% and significance level of *p* < 0.05. One hundred and fifty-four participants completed the survey with a response rate of 96%. From participant responses we found that over 75% of the study population used botanical medicines. Key findings included a diverse repertoire of traditional knowledge on the use of medicinal plants, which included a total of 29 medicinal applications cited across 69 different plant species and 39 families. The most popular species among respondents (irrespective of use) were *Pimenta racemosa* (Mill.) J.W. Moore (FC = 30, RFC = 0.26), *Momordica charantia* L. (FC = 28, RFC = 0.24), *Zingiber officinale* Roscoe (FC = 22, RFC = 0.19) and *Annona muricata* L. (FC = 21, RFC = 0.18). The findings also show the persistence of medico-cultural concepts such as cleansing and cooling, and identified significant associations between the use of botanical medicines and related practices with demographic variables such as education (*p* = 0.05; Fisher’s Exact Test) and health insurance, χ^2^ (1, n = 152) = 4.645, *p* = 0.003. The findings of this study can be used in the identification and archiving of the medicinal plant practices in Barbados and the wider Caribbean, as well as for the larger purposes of biocultural exploration, preservation and further scientific assessment of botanical medicine practices.

## Introduction

The Caribbean region is widely recognized for its rich plant diversity, ranking among the top 6 of 25 global biodiversity hotspots (Carrington, 2007; Cohall, 2014). These plants are utilized extensively throughout the Caribbean in the practice of folklore botanical medicine, which originated from the intense cultural convergence prompted by European colonialism, indigenous cultures of the Americas, the transatlantic slave trade, and immigration of indentured servants from Asia (Bayley, 1949; Feng 1956; Crawford-Daniel and Alexis, 2014). Many of the healing botanical medicines used in the Caribbean today are of West African origin, including the use of *Citrus aurantiifolia* [Christm.] Swingle (Lime)*, Ricinus communis* L. (*Castor* Bean), and *Abrus precatorius* L. (Wild Licorice/Crab’s Eye) (McCaskie, 2017). These were all important species used in West African healing traditions and are still commonly used in Caribbean countries such as Jamaica (Vandebroek and Picking, 2020), Cuba (Heredia-Diaz et al., 2018), the Virgin Islands (Soelberg et al., 2016), and others (Torres-Avilez et al., 2015). However, because of the cultural suppression and syncretism that occurred during the enslavement of West Africans in the Caribbean and after their emancipation, many of the islands differ in respect to their native pharmacopeias (Crawford-Daniel and Alexis, 2014; Sutherland et al., 2014).

Barbados is the easternmost Caribbean island and has a population of 287,000, 95.5% of which are descendants of the enslaved West African people (World Development Indicators, 2019; Jackson et al., 2021). Approximately 65% of the healthcare in Barbados is public and 35% private, however, the public system is universally accessible, free at delivery and, notably, includes access to drugs (Labonte et al., 2017). Despite this, the healthcare expenditure ranks high among other Caribbean or Central American countries, with an increase in disease burden on the island. One in ten Barbadian adults has a chronic, non-communicable disease and the associated mortality rate per 100,000 is increasing (Unwin et al., 2015; Global Burden of Disease Collaborative Network, 2020). Additionally, non-communicable diseases account for 9 of the top 10 causes of death in Barbados (Global Burden of Disease Collaborative Network, 2020).

Unlike its neighboring islands, Barbados was colonized primarily by the British and did not have the influx of indentured servants observed in other southern Caribbean islands (Lane, 1979). While a wealth of literature exists on traditional botanical medicine in larger Caribbean countries (Mahabir and Gulliford, 1997; Merritt-Charles, 2011; Clement et al., 2005), there is a need for more comprehensive studies to be done on this topic in smaller islands such as Barbados, as they were subject to a stronger degree of cultural suppression (Abrahams, 1967). For instance, smaller islands typically had smaller plantations, which meant fewer slaves and greater oversight from the plantation owners, as well as a greater adoption by slaves of the attitudes and expressions of the owners (Abrahams, 1967). This was especially the case amongst the liberated slaves who aspired for a better quality of life, which translated to attaining a British-based education, accessing westernised healthcare, and generally engaging in activities with the British colonial upper-class (Crawford-Daniel and Alexis, 2014). As a result, a gradient developed in the knowledge and use of traditional medicine between the uneducated vs educated, rural vs urban Barbadians (Crawford-Daniel and Alexis, 2014). Additionally, compared to the larger islands, more land mass had to be cultivated to ensure the plantation system was economically viable, which lead both to 1) the loss of hundreds of plant species across the island due to deforestation-many of which were potentially medicinal, and 2) newly freed slaves having few options but to remain on the plantations as labourers after the abolishment of slavery (Bayley, 1949; Watts, 1966; Abrahams, 1967). Currently, the island has approximately 650 species of flowering plants with only two species being endemic (Bayley, 1949; Carrington, 2007; Cohall, 2014). The persistence of many of these outcomes into present-day Barbados makes it a particularly compelling candidate for novel ethnopharmacological studies, as it not only provides information about local botanical medicine practices, but may also elucidate processes that underlie or drive the use of botanical medicine within the region, by allowing for comparisons with larger countries that did not experience the same degree of deculturation.

Even with the loss of some cultural practices and plant species throughout colonization, there are immense health and economic benefits associated with the cultivation, harvesting, extraction, research, and development of plant-based natural health products, as well as potential economic benefit in the marketing of Barbados and the broader Caribbean region as a health and wellness destination, addressing both spiritual and physical health. In fact, the medicinal uses of many of these plants have been investigated and confirmed mainly by *in vitro* and preclinical studies (Cohall, 2014). Therefore, substantial efforts should be made to document and preserve medicinal plant knowledge in the Caribbean and to identify barriers to use. Some such barriers may include the perceived superiority of western medical practices and the resultant attitudes of physicians towards botanical remedies, the possible effects of the unidentified chemical compounds in a plant, and the lack of public knowledge concerning drug-herb interactions.

Therefore, we conducted an observational study in College Lands, St. John, Barbados, to gain insight into the knowledge, attitudes, and practices on the use of botanical medicines in rural communities where the retention of these traditions has been reported to be higher (Crawford-Daniel and Alexis, 2014; Clement et al., 2015), and to determine whether there are factors that might predict or influence the use of these remedies in the surveyed communities. The knowledge gained from this research will be used to identify medicinal plants in the geographical area for further scientific investigation of pharmacological activity, cultivation, and the possible co-modification into effective and commercially viable products, which may help to mitigate some of these main barriers to the use and access of the benefits of botanical medicines.

## Materials and Methods

### Study Setting, Participants, and Ethics Approval

Surveys were administered during April and May of 2018 in three rural communities situated in College Lands, a rural district in the parish of St. John located on the southeastern side of Barbados. These locations included the two communities adjacent to the Codrington College (Latitude/Longitude: 13° 10′ 31″ N/59° 28′ 32″ W) and College Savannah/Consett Bay (Latitude/Longitude: 13° 10′ 60.00″ N/-59° 27′ 59.99″ W). These communities were the sugarcane-producing estates, Society and Consett. They were established in the 17th century by Christopher Codrington, who, after his death, left the estates to be used for the spiritual and physical well-being of people by developing a religious college – known today as Codrington College – while the surrounding lands were left for the people’s livelihoods (Holder, 1988). This bequest was intended to benefit the non-White population of Barbados, including the provision of an education for those who were enslaved; however, slave labour in the estates continued until it was abolished in 1833 (Holder, 1988). This resulted in a unique convergence of slavery and education in an area with a plant biodiversity reflective of ancestral practices and the tropical climate associated with Barbados’ geographical location (Carrington, 2007; Crawford-Daniel and Alexis, 2014).

A map of College Lands was used with census data provided by the administration of the Codrington College and its Trust to assist in locating the community boundaries and the participants. Residents were recruited via written correspondences and flyers which were distributed in the area prior distributing the survey. The survey was randomly administered to persons meeting the eligibility criteria, which required them to be residents of College Lands, St. John, Barbados and at least 18 years of age. The number of surveys administered was based on a sample size of 160, which was calculated at a power of 80%, confidence level of 95%, confidence interval of 4%, and a population size of 216 (as determined from a census conducted in 2018). Surveys were applied and analyzed per person interviewed.

Study approval was obtained from The University of the West Indies (UWI)/Barbados Ministry of Health Ethics Committee/Institutional Review Board (IRB No. 180303-A), and informed consent was obtained prior to administering the survey.

### Survey Instrument

Semi-structured interviews were conducted using a questionnaire designed to probe information regarding the knowledge, attitudes, and practices surrounding the use of botanical medicines, as well as demographic data such as age, gender, education, and annual income. A total of 23 questions were asked, including single-response, multiple-response, and skip logic types. All questions were nominal in nature, excluding age and annual income. This instrument was validated and used in a prior ethnopharmacological study on the island (Cohall et al., 2012). Participants were asked to report information including, but not limited to, any chronic conditions they suffer from, their use of plant-based remedies, the specific plants they use and what they are used for, where they source the medicinal plants from, where they acquired their knowledge of botanical medicine, whether they discuss their use of plant-based remedies with their physician, as well as other non-botanical medicine-related prompts. The survey instrument can be found in Supplementary Data Sheet 1.

### Plant Collection/Identification

Where possible, field officers collected samples of plants which were identified by a plant taxonomist, and vouchered samples were deposited at the herbarium at The University of the West Indies, Cave Hill Campus. In some instances, plants were easily identifiable as crop plants and some recalled as semi-processed products. Lastly, some plants with reported medicinal properties were identified with the use of high resolution pictures from peer-reviewed published sources by the interviewers. The scientific nomenclature of all the plant families and species were verified using the Kew Medicinal Plant Name Services (MPNS) and the final list of plants was reviewed by the plant taxonomist for accuracy.

### Data Analysis

#### Measures

For the purposes of this study, botanical medicine use was determined through participants’ response to the survey question asking “do you use herbs/bush to treat? If no, skip to question 19.” Those citing one or more plants that they personally use in a healing capacity were considered users of botanical medicines and are referred to as “users” in following sections. Those who skipped questions 12–18 were considered “non-users” and referred to as such.

In relation to the primary research question, the knowledge, attitudes, and practices were ascertained through data provided by the users of botanical medicine on the following variables:

• Specific medicinal plants employed in treatment
• Uses/applications of the medicinal plants
• Sources of botanical medicine knowledge
• Sources of medicinal plants
• Communication with doctor about botanical medicine use
• Behaviours and knowledge surrounding drug-herb combination and associated risks
• Recommendation of botanical medicines to others

In relation to the secondary research question, potential predictors of botanical medicine use were identified through cross-tabulation and significance testing of the following variables with “Use of Botanical Medicine” (Y/N):

• Age
• Education Level
• Income
• Physician Relationship
• Parental Use of Botanical Medicine
• Health Insurance (Y/N)

A manual double data entry process was used to enter, verify, and validate the accuracy of data in Microsoft Excel (Microsoft Corporation, Redmond, Washington, United States). All data analysis was performed using SPSS 27 software (SPSS Inc., Chicago, Illinois, United States). The response options for each question were numerically coded beginning at a value of 1. Missing values were assigned a code of “888”, “999”, or were left as “system missing”. 888 codes corresponded to “not applicable” and were assigned if the missing value was from participants who did not identify with the subset of respondents that the question was intended for, and thus were correct in skipping it. 999 codes were labelled “no response” and were used if the missing value was from participants who incorrectly skipped a question that was intended for their specific demographic (e.g., only answers one of a two-part question about their chronic condition). When values were missing from stand-alone questions (e.g., what is your age), they were considered “missing at random” and were left as such.

Ethnobotanical data concerning reported plant species and medicinal uses among the study population were analyzed using the following indices:

##### Use Value

The use value determines the therapeutic versatility of locally known plant species and was calculated using the following formula (Napagoda et al., 2018; Andriamparany et al., 2014; Phillips and Gentry, 1993):

$$\text{UV}=\frac{\Sigma \text{Ui}}{\text{N}}$$

where Ui is the number of uses reported by each informant for a given species and N is the total number of informants. A high use value indicates that the cited species has a diversity of medicinal applications among the participants in the study.

###### Frequency of Citation and Relative Frequency of Citation

The frequency of citation is the number of informants reporting the use of a particular species, while the relative frequency of citation illustrates the local importance of each species among the study’s participants and is given by the following formula (Amjad et al., 2017):

$$\text{RFC}=\frac{\text{FC}}{\text{N}}$$

where FC is the frequency of citation (number of informants reporting the use of a particular species), and N is the total number of informants. This index varies from 0, when nobody cited the use of the plant, to 1 in the case that all informants reported using this species.

All other data (e.g., demographic variables) were summarized using frequencies and percentages. To identify significant associations between variables, contingency tables were created, and Chi-squared analysis was performed. When more than 5 cells had an expected count less than 5, the variables were either recategorized into larger groups, or the Fisher’s Exact Test was used instead of the Chi-squared analysis. If an association was found to have statistical significance (*p* ≤ 0.05), a Z-test of column proportions with Bonferroni correction was carried out to highlight the specific relationships responsible for the significant result. Effect size was measured using Phi (for 2 × 2 tables) or Cramer’s V (for RxC tables).

## Results

### Characteristics of Sample

A total of 154 completed questionnaires were obtained from residents of College Lands, St. John, Barbados with a response rate of 96%. The median age group of respondents was 51–60 years old (*n* = 31, 20.1%), with 58.4% of the sample identifying as female and 41.6% identifying as male. Secondary school was the highest level of education attained by 45.4% of respondents, 30.9% had no higher than a primary level education, and 23.7% had attained an associate degree or higher. Among 136 total respondents, the most reported income range was less than $8,000 BDS per year (*n* = 48, 35.3%) followed by those earning greater than $28,000 BDS per year (*n* = 30, 22.6%). 62.1% of participants (*n* = 95) had at least one chronic condition, the most common of which were Hypertension (*n* = 57, 60.0%), Type II Diabetes Mellitus (*n* = 26, 27.4%), and Arthritis (*n* = 24, 25.3%). The treatment of chronic conditions with prescription medication was reported by 68 of the respondents, however, data was missing for 26 respondents. Participant characteristics are documented in Table 1, and data pertaining to the chronic conditions reported by participants are given in Table 2.

**TABLE 1:** Participant characteristics.

	Total N (%)	Proportion using botanical medicines	
		*n*/N	%
**Age (Years)**			
<20	5 (3.2)	2/5	40.0
21–30	13 (8.4)	8/13	61.5
31–40	20 (13.0)	17/20	85.0
41–50	23 (14.9)	17/23	73.9
51–60	31 (20.1)	24/31	77.4
61–70	29 (18.8)	24/29	82.8
71–80	30 (19.5)	21/30	70.0
81–90	3 (1.9)	3/3	100.0
**Gender**			
Male	64 (41.6)	50/64	78.1
Female	90 (58.4)	66/90	73.3
**Education Level**			
Primary or less	47 (30.9)	41/47	87.2
Secondary	69 (45.4)	52/69	75.4
Associate/Vocational	22 (14.5)	13/22	59.1
Undergraduate	6 (3.9)	4/6	66.7
Graduate	8 (5.3)	5/8	62.5
(Missing values)			
Missing at random	(2)	(1)	
**Annual Income (BDS)** **^a^**			
Less than $8,000	45 (33.8)	34/45	75.6
$8,000-$13,000	18 (13.5)	14/18	77.8
$13,000-$18,000	10 (7.5)	10/10	100.0
$18,000-$23,000	15 (11.3)	13/15	86.7
$23,000-$28,000	12 (9.0)	7/12	58.3
$28,000 **+**	30 (22.6)	19/30	63.3
Retired	3 (2.3)	2/3	66.7
(Missing values)			
Missing at random	(21)	(17)	
**Relationship Status**			
Married	44 (28.6)	33/44	75.0
Single	83 (53.9)	60/83	72.3
Separated	7 (4.5)	7/7	100.0
Divorced	6 (3.9)	6/6	100.0
Widowed	9 (5.8)	6/9	66.7
Common-Law	5 (3.2)	4/5	80.0
**Country of Birth**			
Barbados	147 (95.5)	111/145	76.6
Other Caribbean Country	7 (4.5)	5/7	71.4
**Self-Perceived Health**			
Excellent	9 (5.9)	8/9	88.9
Very Good	29 (19.0)	21/29	72.4
Good	81 (52.9)	60/81	74.1
Fair	29 (19.0)	24/29	82.8
Poor	5 (3.3)	3/5	60.0
(Missing values)			
Missing at random	(1)		
**Chronic Condition**			
Yes	95 (62.1)	72/95	75.8
No	58 (37.9)	43/58	74.1
(Missing values)			
Missing at random	(1)	(1)	
**Parental Use of Botanical Medicines**			
Yes	70 (56.5)	58/70	82.9
No	54 (43.5)	37/54	68.5
(Missing values)			
Missing at random	(30)	(21)	
**Health Insurance**			
Yes	24 (15.8)	14/24	58.3
No	128 (84.2)	101/128	78.9
(Missing values)			
Missing at random	(2)	(1)	
**Type of Healthcare**			
Public	51 (35.9)	43/51	84.3
Private	71 (50.0)	47/71	66.2
Both	20 (14.1)	15/20	75.0
(Missing values)			
Missing at random	(12)		

a 1 BDS = 0.5 USD.

**TABLE 2:** Chronic Conditions and Drugs Prescribed for their Management Among Participants.

Chronic Conditions (*N* = 95)	*n* (%)	Proportion (%) using Botanical Medicines	Drugs Prescribed, *n*		Reported Costs (BDS)^a^ *n*	
Type II Diabetes	26 (27.4)	22/26 (84.6)	Gliclazide	9	Free	6
			Metformin	14	Free	8
			Insulin	7	Free	2
					$27.5/m^b^	1
			Candesartan	1	Free	1
			Sitagliptin-Metformin	2	$104/m	2
Heart Disease	3 (3.2)	2/3 (66.7)	Bisoprolol	2	-	-
			Verapamil	1	Free	1
Hypertension	57 (60.0)	43/57 (75.4)	Valsartan	13	Free	6
			Amlodipine	7	Free	2
					$30/m	1
			Chlorthalidone	2	Free	1
			Telmisartan	3	$40/m	2
			Aspirin	2	Free	1
			Indapamide	4	Free	2
			Bezide (Gliclazide-Metformin)	3	Free	2
				5	Free	2
			Atenolol		$21/m	1
			Verapamil	1	Free	1
			Lisinopril	1	Free	1
			Indapamide-Amlodipine	4	Free	3
					Free	2
			Valsartan-Hydrochlorothiazide	6	< $20/m	1
High cholesterol	16 (16.8)	13/16 (81.3)	Atorvastatin	10	Free	7
			Amlodipine	1	Free	1
			Simvastatin	1	Free	1
			Rosuvastatin	1	Free	1
			Ezetimibe-Simvastatin	1	-	-
Back pain	11 (11.6)	8/11 (72.7)	Acetaminophen	3	Free	2
					≥ $5	1
Arthritis	24 (25.3)	19/24 (79.2)	Diclofenac	3	$6	1
			Acetaminophen	5	Free	2
			Carbamazepine	1	Free	1
			Enalapril	1	-	-
			Indapamide	1	-	-
			Tramadol	1	Free	1
			Glucosamine	1	Free	1
			Methotrexate	2	-	-
			l-ascorbic Acid	1	-	-
			Ferrous Sulphate	2	-	-
			Folic Acid	2	-	-
			Omega 3	1	Free	-
Osteoporosis	1 (1/1)	0	Strontium Ranelate	1	-	-
			Folic acid	1	-	-
			Calcium Carbonate	1	-	-
Sinus	6 (6.3)	6/6 (100.0)	Nasal spray	2	Free	2
Asthma	7 (7.4)	4/7 (57.1)	Albuterol	5	Free	3
			Betamethasone	1	$15	2
			Fluticosone Proprionate-Salmaterol Xinafoate	1	$20	1
			Beclometasone Diproprionate	1	Free	1
			Budesonide-Formoterol	1	Free	1
					Free	1
Neuro/Psychiatric	3 (3.2)	0	Citalopram	1	-	-
			Carbamazepine	1	-	-
			Zolpidem	1	-	-
Heart	2 (2.1)	2/2 (100.0)	Warfarin	1	-	-
Dyspnea	1 (1.1)	1/1 (100.0)	-	-	-	-
Blood/Circulatory	4 (4.2)	2/4 (50.0)	Diosmin	1	$5/m	1
			Ferrous sulphate	1	-	-
Eye	2 (2.1)	1/2 (50.0)	Bimatoprost	1	-	-
			Dorzolamide-timolol	1	$90	1
Cancer	2 (2.1)	1/2 (50.0)	Cyproterone	1	-	-
Thyroid	7 (7.4)	4/7 (57.1)	Levothyroxine	7	Free	2
			Calcium carbonate	1	$12/m	1
					$5	1
					-	-
Autoimmune	1 (1.1)	1/1 (100.0)	-	-	-	-
Kidney	1 (1.1)	1/1 (100.0)	-	-	-	-
GERD	2 (2.1)	2/2 (100.0)	Omeprazole	1	$18	1

a 1 BDS = 0.5 USD.

b m is the abbreviation for month.

### Knowledge, Attitudes, and Practices Surrounding the Use of Botanical Medicines

Results presented in *Characteristics of the Users of Botanical Medicines*, *Plants Used for Medicinal Purposes*, *Acquisition of Knowledge and Sources of Medicinal Plants*, *Discussion of Botanical Medicine Use with Doctor*, *Concomitant Use of Medicinal Plants and Prescription Medication*, and *Recommendation of Botanical Medicines to Others* only pertain to data collected from users of botanical medicines, or simply “users”.

#### Characteristics of the Users of Botanical Medicines

Out of the 154 survey respondents, 116 (75.3%) were users of botanical medicines. The majority of the persons were over 50 years old (62.1%), female (56.9%), had “good” self-perceived health (51.7%), and reported at least one chronic condition (62.1%). Characteristics of the users are provided in Table 1.

#### Plants Used for Medicinal Purposes

As a group, the respondents cited a total of 29 different therapeutic applications across 69 different plants. The FC and RFC indices determined that *Pimenta racemosa* (Mill.) J.W. Moore (Bay Leaf), *Momordica charantia* L. (Cerasee), *Zingiber officinale* Roscoe (Ginger), *Annona muricata* L. (Soursop), *Moringa oleifera* Lam. (Moringa), *Cymbopogon citratus* (DC.) Stapf (Lemongrass), *Aloe vera* (L.) Burm.f. (Aloe), *Persea americana* Mill. (Pear), *Petroselinum crispum* (Mill.) Fuss (Parsley) and *Azadirachta indica* A. Juss. (Neem), were the 10 most cited ethnomedicinal plant species (Table 3) with reference to the respondents who cited the use of these plant species. In terms of the medicinal uses reported for these plants, the UV index identified *A. indica* as the most versatile species (UV = 1.54), followed by *M. charantia* (UV = 1.32), *A. vera* (1.24), *P. racemosa* (1.17), *A. muricata* (1.14) and *Zingiber officinale Roscoe* (1.14), *P. americana* (1.13), *C. citratus* (1.11), *M. oleifera* (1.06) and *P. crispum* (1.06) among the reported citations. Irrespective of plant species, the most common therapeutic applications cited by respondents included “maintenance of health” (*n* = 49), “cooling” (*n* = 34), “hypertension” (*n* = 23), “cough” (*n* = 23), and “cold/flu” (*n* = 18). Table 3 lists all of the plants cited by participants, along with their uses, methods of administration, and FC, RFC, and UV scores.

**TABLE 3:** List of medicinal plants, preparation methods and frequency of administration reported by users (*N* = 116).

Plant				FC (RFC)	UV	Applications		
Family	Species	Voucher No.	Local Name(s)			Reported Uses	Preparation and Administration	Freq. of Treatment
Acanthaceae	*Justicia secunda* Vahl		Bloodroot	2 (0.02)	1.0	Cleansing	Root (dried); decoction	Monthly
						Cold/Flu	Root (dried); decoction	As needed
Amaryllidaceae	*Allium sativum* L		Garlic	9 (0.08)	1.11	Blood/Circulation	Decoction	Bi-weekly
						Joint pain	Leaves; decoction	Weekly
						Cuts	Ingested raw	As needed
						Hypertension	Pods; grinded and ingested with coconut water	Daily
						Immune booster	Infusion	As needed
						Sores	Ingested raw	As needed
Annonaceae	*Annona muricata* L		Soursop	21 (0.18)	1.14	Cooling	Leaves; decoction or infusion	Daily, weekly
						Health maintenance	Leaves; decoction or infusion	Daily, weekly
						Hypertension	Leaves; decoction or infusion	Daily
						*Cancer* prevention	Leaves; decoction	-
						Constipation	Infusion	Daily
						Diabetes	Leaves (dried); decoction	Daily
						Inflammatory conditions	Leaves; decoction	Weekly
	*Annona squamosa* L	DC012	Sugar Apple	4 (0.03)	1.25	Cooling	Leaves; decoction	Daily for 1 week then 2-weeks break
						Health maintenance	Infusion	Daily
						Diabetes	Leaves (dried); decoction	Daily
						Hypertension	Leaves; decoction	Daily
Apiaceae	*Petroselinum crispum* (Mill.) Fuss		Parsley	16 (0.14)	1.06	Hypertension	decoction or infusion	Daily, weekly
						Health maintenance	Decoction	Weekly
						Cooling	infusion	Weekly
						Headache	Infusion	Monthly
						Immune booster	Infusion	As needed
	*Apium graveolens* L		Celery	2 (0.02)	1.0	Hypertension	Decoction or infusion	Daily
						Health maintenance	-	Weekly
	*Foeniculum vulgare* Mill		Fennel	1 (0.01)	2.0	Fever	Infusion	Daily
						Cough	Infusion or inhaled	-
Arecaceae	*Cocos nucifera* L		Coconut	2 (0.02)	1.0	Cooling	Oil (+Epsom salts), applied topically	Every 3 months
						Back pain	Oil, applied topically	Weekly
Asphodelaceae	*Aloe vera* (L.) Burm.f	DC009	Aloe	17 (0.15)	1.24	Health maintenance	Gel; ingested raw, blended with juice, decoction, or infusion	Weekly, monthly
						Cleansing	Decoction or ingested raw	Weekly, monthly
						Cuts	Leaf; cut open, applied topically to wound	As needed
						Cold/Flu	Gel (blended); infusion	As needed
						Cough	Gel; ingested raw	As needed
						Diabetes	Ingested raw	Monthly
						Immune booster	Gel (blended); infusion	As needed
						Purgative	Ingested raw	Weekly
						Sores	Leaf; cut open, applied topically to sore	As needed
Asteraceae	*Chromolaena odorata* (L.) R.M. King and H. Rob		Christmas Bush	4 (0.03)	1.0	Health maintenance	Decoction	Daily
						Cooling	Infusion	Daily
						Fever	Decoction (+Pambaram and Cerasee)	As needed
	*Parthenium hysterophorus* L		Whitehead Bush	2 (0.02)	1.0	Joint pain	Twigs; infusion	As needed
						Eczema	Soaked in water and left on skin until dry	As needed
	*Pluchea carolinensis* (Jacq.) G. Don		Cure-for-All	1 (0.01)	1.0	Cold/Flu	Infusion	As needed
	*Lactuca virosa* Habl		Wild Lettuce	1 (0.01)	2.0	Joint/Back pain	Infusion	Weekly
	*Bidens pilosa* L		Duppy needles/Monkey Needles/Spanish Needles	1 (0.01)	1.0	Hypertension	Decoction	-
Boraginaceae	*Cordia obliqua* Willd	DC006	Clammy Cherry	1 (0.01)	1.0	Cooling	Leaves; decoction	Daily
	*Borago officinalis* L		Starflower	1 (0.01)	1.0	Hormonal balance	Capsule; ingested orally	Daily
	*Symphytum officinale* L		Comfrey	1 (0.01)	1.0	Health maintenance	Decoction or infusion	Daily
Cannabaceae	*Cannabis sativus* L		Marijuana	1 (0.01)	1.0	Vomiting	Leaves; decoction	-
Caricaceae	*Carica papaya* L	DC011	Pawpaw	11 (0.1)	1.27	Hypertension	Decoction, infusion, or ingested raw	Daily, monthly, as needed
						Constipation	Infusion or eaten raw	Daily, as needed
						Cold/Flu	Leaves; decoction	As needed
Clusiaceae	*Clusia plukenetii* Urb		Rock Balsam	1 (0.01)	1.0	Cough	Leaves; chewed	As needed
Crassulaceae	*Kalanchoe pinnata* (Lam.) Pers	DC005	Wonder of the World	11 (0.1)	1.09	Cough	Leaves; decoction or ingested raw	As needed
						Cold/Flu	Dried, decoction	As needed
						Cleansing	Decoction or infusion	Annually
						Headache	Infusion	As needed
						Health maintenance	Decoction or infusion	Daily
						Sores	Applied topically to wound	As needed
Cucurbitaceae	*Momordica charantia* L	DC002	Cerasee	28 (0.24)	1.32	Health maintenance	Dried, decoction or infusion	Weekly, monthly
						Cold/Flu	Decoction	As needed
						Cleansing	Decoction or infusion	Monthly
						Cough	Decoction or infusion	As needed
						Fever	Leaves; decoction or chewed	As needed
						Hypertension	Infusion	Weekly, monthly
						Diabetes	Decoction	As needed, monthly
						*Cancer* prevention	Leaves; decoction	-
						Constipation	Leaves; infusion	Daily
						Cooling	Infusion	-
						Headache	Infusion	As needed
						Upset stomach	Decoction	As needed
	*Cucumis sativus* L		Cucumber	3 (0.03)	1.0	Sore eye	Placed on eyelids	As needed
						Hypertension	Infusion (+parsley)	Daily
	*Citrullus lanatus* (Thumb) Matsum. and Nakai		Melon	1 (0.01)	1.0	Cooling	Rinds; soaked in water and drunk	Daily
Dilleniaceae	*Doliocarpus dentatus* (Aubl.) Standl		Capadulla	1 (0.01)	1.0	Back pain	Bark; decoction	As needed
Ericaceae	*Vaccinium myrtillus L*		Bilberry	1 (0.01)	1.0	Sore eye	Leaves; infusion	As needed
Euphorbiaceae	*Ricinus communis* L	DC004	*Castor* Bean, *Castor* Oil Plant	2 (0.02)	1.5	Sore eye	Oil; applied topically to eye	As needed
						Constipation	Oil; ingested orally	As needed
						Upset stomach	-	-
	*Croton malabaricus* Bedd		Pambaram	2 (0.02)	1.5	Cooling	decoction	Bi-weekly
						Health maintenance	decoction	Daily
						Fever	Decoction (+Christmas Bush and Cerasee)	Daily
Fabaceae	*Caesalpinia pulcherrima* (L.) Sw	DC014	Pride of Barbados, Flower Fence	3 (0.03)	0.67	Cooling	Leaves; decoction	-
	*Abrus precatorius* L		Crab Eye	2 (0.02)	1.0	Health maintenance	decoction	Daily
						Cough	infusion	As needed
	*Senna bicapsularis* (L.) Roxb		*Senna*, Money Bush, Monkey Tamarind	1 (0.01)	1.0	Cleansing	Decoction or infusion	Annually
	*Trigonella foenum-graecum* L		Fenugreek	1 (0.01)	1.0	Joint pain	Infusion	Weekly
Lamiaceae	*Mentha* × *piperita* L./*Mentha* sp		Peppermint, Mint	9 (0.08)	1.0	Cooling	Infusion	Daily, weekly
						Fever	Leaves; decoction	As needed
						Cold/Flu	Infusion	As needed
						Hypertension	Infusion	Daily
						Health maintenance	Infusion	Weekly
	*Ocimum basilicum* L		Basil	3 (0.03)	1.0	Cooling	Infusion	Daily, as needed
	*Thymus vulgaris* L		Thyme	3 (0.03)	0.67	Headache	Infusion	Daily
						Health maintenance	Infusion	Daily
	*Rosmarinus officinalis* L		Rosemary	1 (0.01)	1.0	Cooling	Decoction (+Moringa)	Daily
	*Marrubium vulgare* L		Horehound	1 (0.01)	1.0	Cough	Infusion	As needed
Lauraceae	*Persea americana* Mill		Pear	16 (0.14)	1.13	Cooling	Leaf; decoction or infusion	Daily, weekly
						Hypertension	Leaf; decoction or infusion	Daily
						Health maintenance	Leaf; infusion	Weekly or monthly
						Cold/Flu	Leaf; decoction	As needed
						Diarrhea	Leaf; infusion	Once per week
						Headache	Leaf; infusion	Monthly
						Purgative	Leaf; decoction	Weekly
Linaceae	*Linum usitatissimum* L		Flax, Linseed	1 (0.01)	1.0	Joint pain	Ingested in powder form	Daily
Lythraceae	*Punica granatum* L	DC013	Pomegranate	2 (0.02)	1.0	Cooling	Decoction	-
						Health maintenance	Infusion	Monthly
Malvaceae	*Hibiscus rosa-sinensis* L	DC015	*Hibiscus* Flower	2 (0.02)	1.0	Health maintenance	-	-
Meliaceae	*Azadirachta indica* A. Juss		Neem	13 (0.11)	1.54	Diabetes	Leaf; decoction	Weekly or monthly
						Hypertension	Leaf; decoction	Monthly
						Health maintenance	Decoction	Weekly or monthly
						Cleansing	Leaf; infusion	Weekly
						Cooling	Leaf; infusion	Daily for 1 week then 2-weeks break
						Joint/Back pain	Decoction	Weekly
						Cough	Decoction	As needed
						Cuts	Pods; applied topically to wound	As needed
						Immune booster	Blended, infusion	As needed
						Sore eye	Leaf; decoction	As needed
Moraceae	*Artocarpus altilis* (Parkinson) Fosberg		Breadfruit	3 (0.03)	1.0	Health maintenance	Infusion	Weekly
						Hypertension	Leaf; infusion	Weekly
Moringaceae	*Moringa oleifera* Lam		Moringa	18 (0.16)	1.06	Health maintenance	Leaf; decoction	Daily, weekly
							Nuts; ingested raw	
						Diabetes	Decoction or infusion	Weekly
						Hypertension	Decoction	Weekly
						Cleansing	Decoction (+sugar) or infusion	Weekly, monthly
						Cooling	Decoction	Daily
						Cough	Seeds; chewed	As needed
						Immune booster	Blended, infusion	As needed
Musaceae	*Musa acuminata* Colla	DC007	Banana	2 (0.02)	1.0	Iron supplement	-	-
						Toothache	Stub; decoction	As needed
Myristicaceae	*Myristica fragrans* Houtt		Nutmeg	2 (0.02)	1.0	Upset stomach	Grated, infusion	As needed
						Headache	Mixed with candle grease, applied to cloth, and placed on head	As needed
Myrtaceae	*Syzygium aromaticum* (L.) Merr. and L.M. Perry		Clove	2 (0.02)	1.0	Toothache	Cloves; decoction	As needed
						Health maintenance	-	-
	*Pimenta racemosa* (Mill.) J.W. Moore	DC018	Bay Leaf	30 (0.26)	1.17	Cooling	Leaves; decoction (+sugar) or infusion	Daily, weekly
						Health maintenance	Decoction or infusion	Daily, weekly
						Hypertension	Decoction or infusion	Daily
						Cleansing	Decoction (+sugar)	Monthly
						Cold/Flu	Decoction	As needed
						Headache	Infusion	As needed
						Sinus issues	Decoction	As needed
						Upset stomach	Infusion	Monthly
	*Psidium guajava* L		Guava	1 (0.01)	1.0	Sore throat	Ingested orally	As needed
Oleaceae	*Syringa vulgaris* L		Lilac	1 (0.01)	1.0	-	-	-
Onagraceae	*Oenothera biennis* L		Evening Primrose	1 (0.01)	1.0	Hormonal balance	Capsule, ingested orally	Daily
Papaveracae	*Argemome mexicana* L		Holly Hock	1 (0.01)	1.0	Nosebleed	-	-
Phytolaccaceae	*Petiveria alliacea* L		Gully Root	1 (0.01)	1.0	Cough	Decoction (+sugar and salt)	As needed
Poaceae	*Cymbopogon citratus* (DC.) Stapf	DC001	Lemongrass, Fever Grass	18 (0.16)	1.11	Cough	Decoction	As needed
						Cooling	Decoction	Daily
						Health maintenance	Decoction or infusion	Daily, monthly
						Cold/Flu	-	-
						Fever	Decoction	As needed
						Inflammatory conditions	-	-
						Joint pain	Infusion	Weekly
						Sinus issues	-	-
Ranunculaceae	*Hydrastis canadensis* L		Goldenseal	1 (0.01)	1.0	Health maintenance	-	-
	*Nigella sativa* L		Blackseed, Black Cumin	1 (0.01)	1.0	Hypertension	Oil extract	-
Rubiaceae	*Morinda citrifolia* L		Noni, Dog Dumpling	2 (0.02)	1.0	Joint pain	Leaf, fruit; decoction	As needed
						Health maintenance	Blended with juice	Monthly
	*Psychotria tenuifolia* Sw		Coffee Bush	2 (0.02)	1.0	Health maintenance	decoction	Daily
						Diabetes	Dried, decoction	Daily
Rutaceae	*Citrus limon* (L.) Osbeck		Lemon	4 (0.03)	0.75	Health maintenance	Decoction	Daily
						Cooling	Infusion	-
						Diabetes	Infusion	As needed
	*Dictamnus albus* L		Moses Papa Bush	1 (0.01)	1.0	Cough	-	-
	*Citrus sinensis* (L.) Osbeck		Orange	1 (0.01)	1.0	Immune booster	Blended, infusion Capsule ingested orally	As needed
Salvadoraceae	*Salvadora persica* L		Mustard Bush	1 (0.01)	1.0	Nosebleed	-	-
Scrophulariaceae	*Bontia daphnoides* L		Wild Olive	2 (0.02)	1.0	Health maintenance	Infusion	Daily
						Cooling	Decoction	Daily
Solanaceae	*Solanum tuberosum* L		English Potato	1 (0.01)	1.0	Sore eye	Soak in water, mix with castor oil and use to wash eye	As needed
	*Capsicum annum* L		Cayenne pepper	1 (0.01)	1.0	Sinus issues	-	-
						Diverticulitis	-	-
Verbenaceae	*Lantana camara* L	DC019	Sage	1 (0.01)	0.5	Cooling	Infusion	Daily
						Diabetes	Infusion	Daily
	*Stachytarpheta jamaicensis* (L.) Vahl	DC003	Vervain	5 (0.04)	1.4	Cold/Flu	Ingested orally	As needed
						Cough	Infusion	Weekly
						Hypertension	Decoction or infusion	Weekly
						Joint pain	Infusion	-
						Headache	Infusion	Monthly
						Health maintenance	Infusion	Monthly
Zingiberaceae	*Zingiber officinale* Roscoe		Ginger	22 (0.19)	1.14	Health maintenance	Rhizome (grated or whole); decoction or infusion	Daily, weekly, monthly
						Joint/Back pain	Rhizome; decoction, infusion, or cooked in stew	Daily, weekly
						Blood/Circulation	Rhizome; decoction	Bi-weekly
						Hypertension	Rhizome; decoction	Weekly
						Upset stomach	Rhizome; decoction or infusion	As needed
						Cooling	Infusion	Daily
						Diarrhea	Rhizome; decoction	As needed
						Dyspnea	Infusion	Daily
						Immune booster	Blended, infusion	As needed
						Inflammatory conditions	-	-
						Sinus issues	-	-
	*Curcuma longa* L		Turmeric	11 (0.1)	1.27	Health maintenance	Decoction, infusion or tablet ingested orally	Daily, weekly, monthly
						Hypertension	Decoction or infusion	Daily, weekly
						Joint/Back pain	Decoction, infusion, or cooked in stew	Daily, weekly
						Blood/Circulation	Decoction	Bi-weekly
						Inflammatory conditions	Decoction	As needed

#### Acquisition of Knowledge and Sources of Medicinal Plants

For most of the respondents (*n* = 92), botanical medicine knowledge was acquired from family members. Friends were the second most frequent source of knowledge (*n* = 41), followed by the internet (*n* = 21), books (*n* = 11), and colleagues (*n* = 5). Pharmacists, the elderly, and a health and beauty company were cited by only one respondent each. None of the respondents reported acquiring any knowledge from doctors.

**FIGURE 1:**
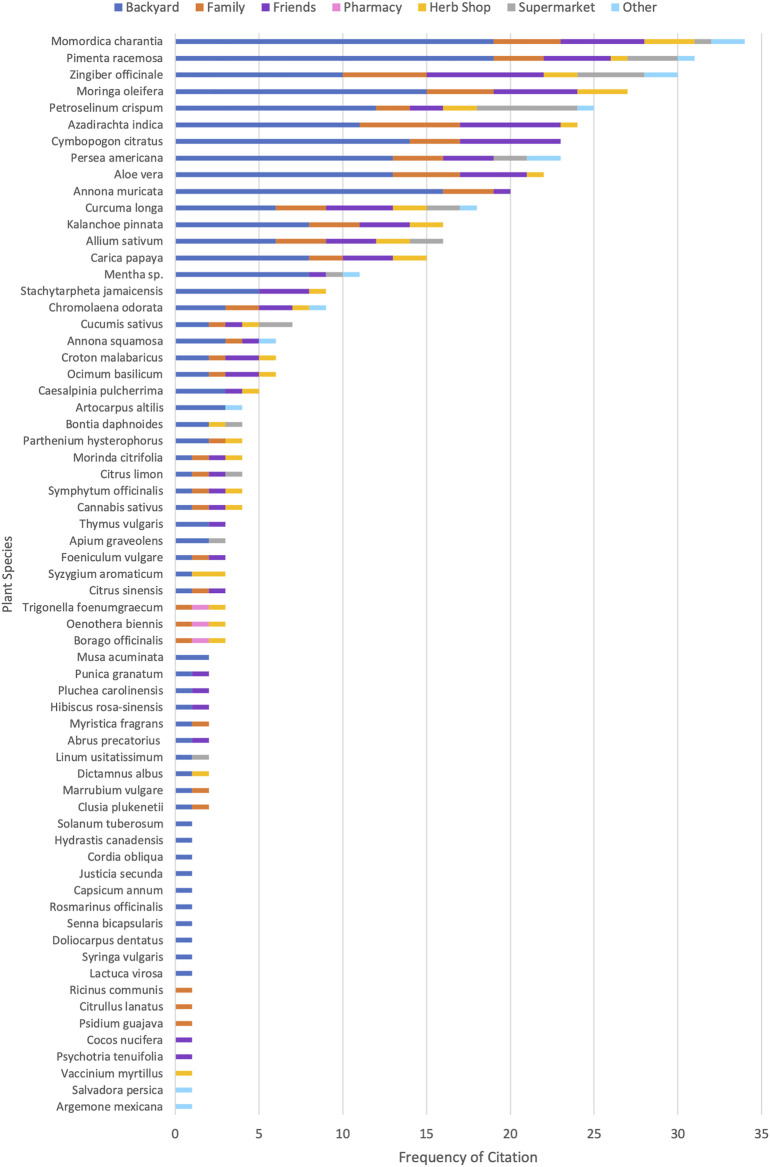
Sources of acquisition of specific medicinal plants as cited by users. Frequency of citation reflects the number of respondents acquiring that species from the given source and includes cases in which respondents cited one species multiple times with different sources.

Most respondents (*n* = 79) retrieved medicinal plants from their backyard, although they were also commonly acquired from friends (*n* = 28), family (*n* = 20) and, to a lesser extent, natural product shops (*n* = 13) and the supermarket (*n* = 13). Only one respondent obtained medicinal plants from the pharmacy. Other sources cited by respondents included the community (*n* = 2), town (*n* = 2), gully (*n* = 2), market (*n* = 1), and neighbours (*n* = 1). Figure 1 provides the specific plants acquired from each source.

Significant associations were found between the acquisition of knowledge and the source of medicinal plants. Respondents who acquired botanical medicine knowledge from family members were significantly more likely to source their medicinal plants from family than those who did not learn from their family members (22.5 vs 0.0%) (*p* = 0.02; Fisher’s Exact Test) (Table 4). Similarly, respondents who acquired their knowledge from friends were significantly more likely to source medicinal plants from friends compared to those who learned from other sources (41.7 vs 18.1%), χ^2^ (1, *n* = 108) = 6.967, *p* = 0.008) (Table 5). The respective effect sizes were 0.22 (Phi) and 0.25 (Phi).

**TABLE 4:** Contingency Table and Fisher’s Exact Test Results for Knowledge Acquired from Family x Medicinal Plants Sourced from Family (n = 108).

Medicinal Plants from Family	Knowledge from Family		*Row Total (%)*	
	Yes	No		*p* **-value (ES)**
Yes	20 (22.5)^a^	0 (0.0)^a^	20 (18.5)	
No	69 (77.5)^a^	19 (100.0)^a^	88 (81.5)	0.02 (0.22)
***Column Total (%)***	89 (100.0)	19 (100.0)	108 (100.0)	

a Percent within *Knowledge from Family* (column %).

*Note:* ES = effect size.

**TABLE 5:** Contingency Table and Chi-Squared Results for Knowledge Acquired from Friends x Medicinal Plants Sourced from Friends (*n* = 108, df = 1).

Medicinal Plants from Friends	Knowledge from Friends		*Row Total (%)*	χ^2^, *p*-value (ES)
	Yes	No		
Yes	15 (41.7)^a^	13 (18.1)^a^	28 (25.9)	
No	21 (58.3)^a^	59 (81.9)^a^	80 (74.1)	6.967, 0.008 (0.25)
***Column Total (%)***	36 (100.0)	72 (100.0)	108 (100.0)	

a Percent within *Knowledge from Friends* (column %).

*Note:* ES = effect size.

The source of medicinal plants was also significantly associated with education, and with annual income. Participants with a primary level education were more likely to acquire medicinal plants from their backyard than were people with a secondary level education (89.5 vs 61.7%) (*p* = 0.02; Fisher’s Exact Test) (Table 6), and participants earning over $18,000 per year were more likely to acquire the plants from their family members compared to those earning less than $18000 BDS per year (29.4 vs 10.7%), χ^2^ (1, *n* = 90) = 5.060, *p* = 0.02) (Table 7). The effect sizes were 0.28 (Cramer’s V) and −0.24 (Phi), respectively.

**TABLE 6:** Contingency Table and Fisher’s Exact Test Results for Education Level x Medicinal Plants Sourced from Backyard (*n* = 107).

Level of Education	Medicinal Plants from Backyard		*Row Total (%)*	
	Yes	No		*p* **-value (ES)**
Primary or less	34 (89.5)^a^	4 (10.5)^a^	38 (100.0)	
Secondary	29 (61.7)^a^	18 (38.3)^a^	46 (100.0)	
Associate/Vocational	9 (69.2)^a^	4 (30.8)^a^	13 (100.0)	0.02 (0.28)
Undergraduate **+**	6 (66.7)^a^	3 (33.3)^a^	9 (100.0)	
***Column Total (%)***	78 (72.9)	29 (27.1)	107 (100.0)	

a Percent within *Level of Education* (row %).

*Note:* ES = effect size.

**TABLE 7:** Contingency Table and Chi-Squared Results for Annual Income x Medicinal Plants Sourced from Family (n = 90, df = 1).

Annual Income	Botanical Medicines from Family		*Row Total (%)*	χ^2^, *p*-value (ES)
	Yes	No		
Less than $18,000	6 (10.7)^a^	50 (89.3)^a^	56 (100.0)	
More than $18,000	10 (29.4)^a^	24 (70.6)^a^	34 (100.0)	5.060, 0.02 (0.24)
***Column Total (%)***	16 (17.8)	74 (82.2)	90 (100.0)	

a Percent within *Annual Income* (row %).

*Note:* ES = effect size.

#### Discussion of Botanical Medicine Use With Doctor

Of the 103 users for which valid data were available, 80 (77.7%) indicated that they do not discuss the use of botanical medicines with their doctor. Most respondents who had discussed this topic with their doctor also reported them as having a positive attitude towards it (*n* = 15, 62.5%).

#### Concomitant Use of Medicinal Plants and Prescription Medication

Out of the 104 users with valid responses, 32 (30.8%) reported using medicinal plants alongside prescription drugs, while the remaining 72 (69.2%) did not combine the two. Reasons for abstaining were provided by 33 participants: 18 (54.5%) cited interaction effects, 9 (27.3%) didn’t take prescription medication at all, 3 (9.1%) believed botanical medicines to be the superior treatment, 2 (6.1%) wanted to ascertain the efficacy of botanical medicines alone, and 1 (3.0%) reported taking them each at different times. In terms of risk awareness, 40% of respondents (*n* = 28) reported being aware of the possible risks associated with herb-drug interactions.

Concomitant use of medicinal plants and prescription drugs was significantly less practiced among participants whose parents are users of botanical medicines (80.0 vs 60.6%) χ^2^ (1, *n* = 88) = 3.911, *p* = 0.05, as well as among participants with no chronic conditions (88.2 vs 59.4%) χ^2^ (1, *n* = 103) = 8.831, *p* = 0.003 (Table 8). The respective effect sizes were 0.21 (Phi) and 0.29 (Phi). Additionally, concomitant use was more frequent among respondents who did not discuss botanical medicines with their doctors (*n* = 19) than among those who did discuss with their doctors (*n* = 11), however, this result was marginally insignificant (26.8 vs 47.8%) χ^2^ (1, *n* = 94) = 3.548, *p* = 0.06.

**TABLE 8:** Contingency Table and Chi-Squared Results for Parental Use of Botanical Medicines (*n* = 88, df = 1) and Chronic Condition (*n* = 103, df = 1) x Concomitant Use of Conventional and Botanical Medicines.

	Concomitant Use of Botanical and Conventional Medicines		*Row Total (%)*	χ^2^, *p*-value (ES)
	Yes	No		
**Parental Herb Use**				
Yes	11 (20.0)^a^	44 (80.0)^a^	55 (100.0)	
No	13 (39.4)^a^	20 (60.6)^a^	33 (100.0)	3.911, 0.05 (0.21)
***Column Total (%)***	24 (27.3)	64 (72.7)	88 (100.0)	
**Chronic Condition**				
Yes	28 (40.6)^b^	41 (59.4)^b^	69 (100.0)	
No	4 (11.8)^b^	30 (88.2)^b^	34 (100.0)	8.831, 0.003 (0.29)
***Column Total (%)***	32 (31.1)	71 (68.9)	103 (100.0)	

a Percent within *Parental Use of Botanical Medicines* (row %).

b Percent within *Chronic Condition* (row %).

*Note:* ES = effect size.

#### Recommendation of Botanical Medicines to Others

88.3% of respondents (*n* = 88) reported that they would recommend the use of botanical medicines to someone else.

### Predictors of Medicinal Plant Use

A significant association was found between education and medicinal plant use. Respondents with a primary level education were significantly more likely to use medicinal plants compared to those with an associate/vocational degree (87.2 vs 59.1%) (*p* = 0.05; Fisher’s Exact Test) (Table 9). The effect size was 0.2 (Cramer’s V).

**TABLE 9:** Contingency Tables and Fisher’s Exact Test/Chi-Squared Results for Level of Education (*n* = 152, df = 3) and Health Insurance (*n* = 152, df = 1) x Botanical Medicine Use.

	Use of Botanical Medicines		*Row Total (%)*	*p*-value (ES)
	Users	Non-Users		
**Level of Education**			
Primary or less	41 (87.2)^a^	6 (12.8)^a^	47 (100.0)	*p* = 0.05 (0.23)
Secondary	52 (75.4)^a^	17 (24.6)^a^	69 (100.0)	
Associate/Vocational	13 (59.1)	9 (40.9)	22 (100.0)	
Undergraduate **+**	9 (64.3)	5 (35.7)	14 (100.0)	
***Column Total (%)***	115 (75.7)	37 (24.3)	152 (100.0)	
**Health Insurance**				**χ^2^, *p*-value (ES)**
Yes	14 (58.3)^b^	10 (41.7)^b^	24 (100.0)	4.645, 0.003 (0.20)
No	101 (78.9)^b^	27 (21.1)^b^	128 (100.0)	
***Column Total (%)***	115 (75.7)	37 (24.3)	152 (100.0)	

a Percent within *Level of Education* (row %).

b Percent within *Health Insurance* (row %).

*Note:* ES = effect size.

Health insurance was also significantly associated with medicinal plant use. Participants who did not have health insurance were more likely to use medicinal plants than those with insurance (58.3 vs 78.9%) χ^2^ (1, *n* = 152) = 4.645, *p* = 0.03 (Table 9). The effect size was -0.2 (Phi).

Medicinal plant use was more frequently reported by respondents whose parents also use medicinal plants (*n* = 58), compared to those whose parents do not (*n* = 37); however, this result was marginally insignificant (82.9 vs 68.5%) χ^2^ (1, *n* = 124) = 3.498, *p* = 0.06.

No significant relationships were found between the use of medicinal plants and age (55.6 vs 79.1% vs 80.0 vs 72.7%; *p* = 0.19, Fisher’s Exact Test), annual income (75.0 vs 85.7% vs 74.1 vs 63.3%; χ^2^ [1, *n* = 133] = 3.825, *p* = 0.28), nor physician relationship (74.6 vs 86.7%; *p* = 0.52, Fisher’s Exact Test).

## Discussion

The primary purpose of this study was to determine the extent of use of botanical medicines by persons residing in three rural communities in College Lands, St. John, Barbados, and to gain insight into the knowledge, attitudes, and practices surrounding plant-based medicines in this locale. Secondary to this was the identification of potential factors that may influence the use of botanical medicines in that geographical location. The survey determined that over 75% of the study participants used plants for medicinal purposes, with the majority being female and over the age of 50. A diverse repertoire of medicinal plant knowledge was identified, both in terms of the specific plants used by respondents, as well as the multitude of conditions for which these plants were used. A total of 69 plant species belonging to 39 botanical families were reported, the most dominant being Lamiaceae (6 species) and Asteraceae (5 species). The high prevalence of botanical medicine use is expected considering the rural location of the study and thus these findings cannot be generalized to the whole population. In fact, other studies conducted in Barbados found that only ∼30% of the whole Barbados population uses botanical medicines (Cohall et al., 2012; Peter, 2013). The low prevalence reported in these studies could partly be explained by the widespread adoption of Western medicine and practices, but could also be related to the deforestation that occurred in Barbados to make space for sugar cane plantations, which ultimately caused the number of plant species to diminish significantly and thus left few species to be used medicinally (Bayley, 1949). *P. racemosa* (Bay Leaf), *M. charantia* (Cerasee), *Zingiber officinale Roscoe* (Ginger), and *A. muricata* (Soursop) were the most frequently cited plants and were among those with the highest UV scores, indicating both the local importance and therapeutic versatility of these species within the study’s population at the time of the survey. Although recent ethnobotanical studies are generally lacking in Barbados, a survey conducted in 2013 found similar results regarding the plant species used by participants and their medicinal applications (Peter, 2013). This study identified 93 plant species that were cited as useful, and approximately 41% of those species were common to those identified in this survey. Many of the same use reports for plant species were highlighted in both surveys, such as the use of *A. indica* (Neem) for diabetes and hypertension, or *Kalanchoe pinnata* [Lam.] Pers. (Wonder-of-the-World) for cough, colds/flu, and cleansing. However, our study highlighted novel use reports for some of the most reported species in both studies, including *M. charantia,* which our study identified as useful for cancer prevention, constipation, cooling, headache, and upset stomach, as well as *C. papaya* (Pawpaw), which was identified as useful in both colds/flu and constipation. Notably, there were no similarities in the use reports for *Stachytarpheta jamaicensis* (L.) Vahl (Vervain) identified by Peter (2013)-which were few and mainly neurological - and those identified in this study, which were more diverse and included cough, colds/flu, hypertension, joint pain, and health maintenance. This finding is noteworthy considering the much smaller and more focused rural setting of this study compared to that done by Peter, which surveyed 11 parishes across Barbados. In particular, this may provide support for the notion that traditional plant knowledge is retained and preserved to a greater extent in rural communities than in more urban localities.

Hypertension, cough, and influenza proved to be the top three medical illnesses that participants in our study treated with botanical medicines, which is not surprising considering hypertension, heart disease and lower respiratory infections are among the top ten causes of morbidity and mortality in Barbados (Global Burden of Disease Collaborative Network, 2020). Our study also showed that 62.1% of medicinal plant users suffered from at least one chronic non-communicable condition, with hypertension being the most prevalent. This is not alarming considering the median age group was 51–60 years, a popular age group for the onset of degenerative chronic diseases as well as a high prevalence of chronic conditions in Barbados (Unwin et al., 2015; Prasad et al., 2012). In fact, the 2015 Health of the Nation Survey found that roughly 45% of Barbadian adults are hypertensive, 25% have diabetes, and 21% are hypercholesterolemic; additionally, these figures were shown to increase with age (Unwin et al., 2015). Interestingly, botanical medicines were most frequently used for “health maintenance”, cleansing (i.e., detoxifying), and cooling (i.e., eliminating heat or irritable behaviour), which are folkloric practices. It is likely that these practices persist in present-day Barbados as relics of the historical beliefs concerning health and disease that underpinned the traditions held by the enslaved West Africans and European colonialists. For example, two pillars of West African health and healing traditions include spiritual cleansing, as well as achieving and maintaining stability among the physical, mental, spiritual, and emotional domains – both of which involve the use of medicinal plants (White, 2015). The use of “cleansing” remedies in Caribbean society today echoes this spiritual cleansing practiced by slaves, although it has gradually become more focused on physical rather than extra-physical healing (Clement et al., 2015; White, 2015). Similarly, the botanical medicines used for “health maintenance” today are likely to reflect those used by West African slaves to achieve stability among the physical, mental, and spiritual domains. The use of cooling remedies, however, mirrors the European humoral theory of health and disease in which diseases were classified as hot or cold – the former being treated with cooling remedies and vice versa (Cook and Walker, 2013).

Other ethnobotanical studies conducted in the Caribbean basin have also documented the use of medicinal plants for cleansing and cooling (Clement et al., 2015; Picking et al., 2015; Tareau et al., 2017), supporting the notion that these medico-cultural concepts represent a lasting impact of the cultural diversity that began in the Caribbean during the colonial era. However, the most compelling support of this finding is provided by Handler and Jacoby (1993) in their examination of slave medicine and plant use, in which the medicinal plants used by slaves prior to 1834 are documented. The list cites several plants that are still used in Barbados today (as identified by this study and Peter (2013)), as well as the medico-cultural uses of these plants to “purify” or “cleanse” the body or protect against “poisons of a hot nature.” While the former is likely a reflection of West African traditional remedies as discussed above, the latter implies the adoption of European healing beliefs by the slave population and the development of novel pharmacopeias in the region. From a clinicopathological viewpoint, cleansing and cooling remedies could have been used extensively during the period of enslavement due to prevalence of the tropical infectious conditions in the West Indies which affected the inhabitants of the islands. The coast of West Africa, the common site of the embarking of slaves to vessels en route to the Caribbean, was considered a breeding ground for infectious diseases which led to the spread of these conditions in the West Indies (Handler, 2006).

Many of the plant use reports highlighted in this study overlap with those reported in ethnobotanical studies conducted in West Africa and Europe. *M. oleifera* (Moringa), which informants in this study reported to be useful for hypertension, cough, and diabetes, has been documented for the same traditional uses in both Benin and Ghana (Oppong Bekoe et al., 2020; Agoyi et al., 2014). Additional parallels are seen with *A. indica* (Neem), which is used for diabetes and anti-bacterial purposes both in Nigeria and in Barbados, as demonstrated by our findings (Lifongo et al., 2014). In Europe, on the other hand, similar use reports have been found for 1) *P. racemosa* (Bay Leaf), including headache, upset stomach, and colds, 2) *M. charantia* (Cerasee), including upset stomach, 3) *Mentha* x *piperita* L. (Peppermint), including colds/flu, and 4) *Aloe vera* Burm.f. (Aloe), including purgative, cuts and sores (Polat and Satil, 2012; Brussell, 2004; Gonzalez et al., 2010; Leonti et al., 2010). A study carried out in Trinidad and Tobago, a larger island in the south of the Caribbean, has also identified similar botanical families and plant species which can be found in Barbados and were reported to have medicinal properties related to ancestral practices. *Stachytarpheta jamaicensis* (L.). Vahl, *Senna alata* (L.) Roxb. and *Momordica charantia* L. were reported to be widely used for cooling/cleansing in that territory (Clement et al., 2015).

The preponderance of botanical medicine users acquiring knowledge from family members could be related to the rurality of the College Lands’ communities. In the post-emancipation era, many liberated slaves migrated to urban centers in pursuit of a better education and achieving middle-class respectability, which meant conforming to British sociopolitical standards. As a result, traditional healing knowledge was largely confined to those who remained in the rural communities (Proctor, 1980). Therefore, the knowledge and use of medicinal plants by persons residing in these communities were likely passed down to them from older generations. This also supports our finding that people with a primary level education were more likely to source medicinal plants from their backyard as compared to those with a secondary level education. In fact, the adoption of western practices, including medicine, by the liberated slaves and their descendants who pursued higher education meant that traditional healing knowledge was essentially relegated the poorer and uneducated population (Proctor, 1980). Studies carried out in Jamaica and Trinidad have found interesting results concerning the relationship between education and the use of botanical medicines. In these studies, the respective prevalence of medicinal plant use among participants was inversely proportional to level of education attained; use was consistently lowest among participants with tertiary education (i.e., post-secondary), and the highest among participants with little to no education (Mahabir and Gulliford, 1997; Merritt-Charles, 2011; Picking et al., 2011). One study investigated the factors affecting the decision to use botanical medicines among asthmatic patients in Trinidad; interestingly, Garlic (*Allium sativum* L.) and Echinacea (*Echinacea purpurea* (L.) Moench) were the preferred plant species among users with higher levels of education – both of which have strong scientific evidence supporting their medicinal properties (Clement et al., 2005). In our study, significant differences in the use of botanical medicines were observed between participants with a primary level education and those with associates/vocational degrees. While this is similar to the general trends described above, no difference in use was observed between primary and tertiary (undergraduate/graduate degree) level participants. However, this could be the result of a small sample size or a lack of participants having a tertiary education level in our sample. Additionally, respondents who did not have health insurance were more likely to use botanical medicines than those with health insurance which further supports the socio-economic divide on the use of botanical medicines observed in the Caribbean territories. No significant associations were observed with annual income and the participants that used botanical medicines in this study. However, a modest number of persons identified as users earned an annual income greater than BDS $28,000.00 (*n* = 19). This supports a similar trend identified in Trinidad and Tobago where persons in the higher socio-economic class were exposed to the benefits of medicinal plants, but this may be limited to co-modified plant products with strong scientific evidence supporting their medical uses (Clement et al., 2005).

Our finding that combining medicinal plants and prescription medications was less common among people whose parents use medicinal plants could indicate that these participants were mirroring the practices they observed from their parents, whose traditional knowledge and experience may have elucidated some potential risks associated with those practices. A similar element of teaching/learning could be responsible for the observation that the concomitant use of botanical and conventional medicines was less frequent among people who discussed botanical medicines with their doctor. Although the association between age and the use of botanical medicine was not significant in our study, findings from other studies provide support for a general, cross-cultural trend of greater medicinal plant use and knowledge among older individuals and females, as observed in our study (Longuefosse and Nossin, 1996; Begossi et al., 2002; Quinlan and Quinlan, 2007). While the former could be interpreted as evidence of knowledge degradation through the younger generations, the latter may be explained by the notion that women are typically the principal caretakers and health custodians of the household, and in general seem to be more observant of their health than men. In fact, several studies have found that women are more likely to carry out their own forms of health management than are men (Liang et al., 1999; Turner and McClure, 2003; Stjernberg and Berglund, 2005). Interestingly, most participants reported earning an annual income, which may suggest that most of the sample may be employed. Thus, the relatively low gender disparity in botanical medicine use could be the result of more women being in the workforce rather than the sole traditional caretaking role in the household.

The 1969 Health Services Act of Barbados, Cap. 44 and the Drug Services Act 1980 provide the framework which ensures public access to quality drugs under specific categories, especially drugs for chronic non-communicable conditions (CNCDs), at affordable prices to citizens and permanent residents regardless of their socio-economic circumstances (Hennis et al., 2005). As observed in Table 2, persons who reported having one or more CNCDs (*n* = 95) had access to free or reasonable pharmaceutical products to treat their conditions and 75.8% of these persons were reported users of botanical medicines. This highlights an integration of traditional and westernized healing practices by these individuals as a result of cultural syncretism which may be irrespective of the cost of accessing health care. The lack of association found between the use of botanical medicines and the nature of doctor-patient relationships is also interesting due to the majority of medicinal plant users not discussing this practice with their physicians. It could be that these individuals have internalized the stigma surrounding the use of botanical medicines and, in turn, do not discuss it with their physician for fear of being judged or ridiculed. While the majority of respondents would recommend the use of botanical medicines to their peers and family, only a minority were aware of risks associated with the use of these plants as medicines.

These findings support the need for more public education and awareness campaigns on the safe use of botanical medicines. It also highlights the need to archive information about the traditional uses of botanical medicines in a territory with low endemic plant species and diversity coupled with a relatively high burden of medical conditions. While the findings from this study are valuable, there are some limitations. The study data was collected by semi-structured interviews using a validated questionnaire and is likely to be affected by recall bias by the participants. This was evident due to differences in the accuracy and completeness of the recollections retrieved from the study participants on their knowledge and practices on the use of botanical medicines. Also, the use value (UV) and relative frequency of citation (RFC) are dynamic and will vary with changes in locations, the knowledge among the study participants and other factors unique to the study population and their practices. UV determines the extent to which a species can be used; thus, species with a high UV are more exploited in the study area to treat a particular ailment than those with a low UV (Amjad et al., 2017). Data have been analyzed and discussed to highlight comparisons within the dataset and other studies, but this may be limited because of variance and thus should be interpreted with caution. Further, this study was also done in a rural area of Barbados and while these findings may be generalizable to other rural Caribbean areas, they are not generalizable to the whole island of Barbados or urbanised areas of the Caribbean. Finally, field officers collected samples of the plants where possible and these were identified by plant taxonomist at the herbarium at the University of the West Indies, Cave Hill Campus. The identification of the other medicinal plants was by their recognition as crop plants and others were identified by the use of high-resolution photographs of the plants and by the informants’ recall. To a lesser extent, some plants were reported as being sourced as semi-processed products, for example, *Lactuca virosa* Habl. (Wild Lettuce), *Oenothera biennis* L. (Evening Primrose)*,* Linum *usitatissimum* L. (Linseed) and *Borago officinalis* L. (Starflower). Unfortunately, product details inclusive of batch numbers were not able to be retrieved by the interviewers.

## Conclusion

The objectives of this study were achieved by the detailed documentation of the knowledge, attitudes, and practices of botanical medicines in rural Barbados, particularly in a district that has never been studied before. Demographic and socioeconomic variables which influence the use of botanical medicines in the surveyed communities were also identified. Additionally, our survey showed the persistence of medico-cultural concepts such as “cooling” and “cleansing” as well as the use of globally recognizable plants, some with West African origins. These findings demonstrate the permeation of ancestral healing practices to persons in these rural communities. Efforts must be mobilized to archive these practices for sensitizing the wider Barbadian and Caribbean population where the use of botanical medicines is much lower. The identification of practices and factors that may influence the use of botanical medicines can promote larger-scale studies to help determine how to overcome certain barriers to the use of medicinal plants, as well as to appropriately establish safety and efficacy studies to further evaluate the pharmacological effects of traditional plant-based remedies.

## Data Availability

The raw data supporting the conclusions of this article will be made available by the authors.
