# Supplementary material for: Knowledge, Attitudes and Practices on the Use of Botanical Medicines in a Rural Caribbean Territory
Source: Front Pharmacol. 2021 Oct 27;12:713855. doi: 10.3389/fphar.2021.713855 (PMC8579079; doi:10.3389/fphar.2021.713855)
Supplement: Supplementary file 1 [file DataSheet1.docx]

**Questionnaire – Medicinal Plants of College Lands, St. John**

1. Circle your gender: male / female

2. Check the highest year of school completed

 Primary School

 Secondary school

 Associate degree: Technical

 Undergraduate

 Graduate

3. Are you currently?

 Married

 Single

 Separated

 Divorced

 Widowed

4. Age:

 Less than 20

 21-30

 31-40

 41-50

 51-60

 61-70

 71-80

 81-90

 90+

5. Country of Birth:

 Barbados

 U.S

 U.K

 Other Caribbean country (specify) ___________________________

 Other: ______________________

6. What is your annual income in Bajan $?

 Less than $8 000

 $8 000-13 000

 13 000-18 000

 18 000-23 000

 23 000-28 000

 28 000+

**7**. In general, would you say your health is?

 Excellent

 Very good

 Good,

 Fair

 Poor

8. Do you currently suffer from a chronic condition? (If No, skip to number 11)

 Yes

 No

9. If yes, check any that apply:

| Name of the chronic condition | Name of the prescribed drugs(by the doctor) | How much ($) does this prescription drugs cost |
| --- | --- | --- |
| - Diabetes type 1 - Diabetes type 2 - Heart disease - Lung Disease - High cholesterol - High blood pressure - Back Pain - Arthritis - Other_________________ - ______________________ - ______________________ | ____________________  ____________________  ____________________  ____________________  ____________________  ____________________  ____________________  ____________________  ____________________  ____________________  ____________________ | ____________________  ____________________  ____________________  ____________________  ____________________  ____________________  ____________________  ____________________  ____________________  ____________________  ____________________ |

10. Do you take any herbs/bushto treat your chronic condition that is not prescribed by a physician?

- Yes
- No

11. Do your parents take any herbs/bush that is not prescribed by a physician?

- Yes
- No

12. Do you take Bush/herbs to treat? Check any that apply to you **(If No, skip to question 19)**

| Do you take bush/herbs to treat? | What is the name of this bush/herb? | How do you prepare it for use? | How often do you take it? (Annually/ Monthly/Weekly/Daily) |
| --- | --- | --- | --- |
| - Fever - Diarrhoea - Cooling - Headache - Toothache - Maintenance of health condition - Eczema - Sore eye - Diabetes (Sugar) - High Blood Pressure - Athlete’s foot - Constipation - Vomiting - Cough - Joint pain - Back pain - Cuts - Sores - Other | ____________________  ____________________  ____________________  ____________________  ____________________  ____________________  ____________________  ____________________  ____________________  ____________________  ____________________  ____________________  ____________________  ____________________  ____________________  ____________________  ____________________  ____________________  ____________________ | ____________________  ____________________  ____________________  ____________________  ____________________  ____________________  ____________________  ____________________  ____________________  ____________________  ____________________  ____________________  ____________________  ____________________  ____________________  ____________________  ____________________  ____________________  ____________________ | ____________________  ____________________  ____________________  ____________________  ____________________  ____________________  ____________________  ____________________  ____________________  ____________________  ____________________  ____________________  ____________________  ____________________  ____________________  ____________________  ____________________  ____________________  ____________________ |

13. Do you talk about your bush/herbs with your doctor?

- Yes
- No

14. ***If yes***, how did your doctor react?

- Positively
- Negatively

15. General Question about your Bush/herb: Check any that apply

| How did you learn about this herbs/bush | Where did you get it | Do you take it by capsule or loose herb |
| --- | --- | --- |
| - Family - Friends - Colleagues - Books - Internet - Doctor - Pharmacist - Other_____________ | - Backyard - Supermarket - Pharmacy - Herb Shop - Friends - Family - Other ___________ - ________________ | - Capsule - Loose herbs - Jelly |

16. Do you take this herbs/bush with prescribed drugs?

- Yes
- No

17. ***If no***, why (e.g.: possible interaction)

_______________________________

18. ***If yes***, are you aware of the possible risks involved with the interaction of the medication from the doctor and the herbs? Check the right one

- Yes
- No

19. Do you have a good relationship with your physician? Check the right one

- Yes
- No

20. Do you have health insurance?

- Yes
- No

21. Do you go to a private or public doctor?

- Private
- Public

22. **If you are taking herbs/bush**, would you recommend this drug to someone else?

- Yes
- No

23. ***If no***, why not?

______________________________________________

______________________________________________
